# A Comprehensive Review of the Endometrial Receptivity Array in Euploid Embryo Transfer Cycles

**DOI:** 10.7759/cureus.63173

**Published:** 2024-06-26

**Authors:** Medhavi Sharma, Pankhuri Dubey, Urmila Sunda, Hard Tilva

**Affiliations:** 1 Obstetrics and Gynaecology, All India Institute of Medical Sciences, Rajkot, IND; 2 Obstetrics and Gynaecology, Jawaharlal Nehru Medical College, Datta Meghe Institute of Higher Education and Research, Wardha, IND

**Keywords:** personalized embryo transfer timing, gene expression profiling, implantation window, assisted reproductive technology (art), euploid embryo transfer, endometrial receptivity array (era)

## Abstract

The Endometrial Receptivity Array (ERA) is a revolutionary molecular diagnostic tool that determines the optimal timing for embryo transfer by analyzing the gene expression profile of endometrial tissue. This comprehensive review examines the significance and application of ERA in euploid embryo transfer cycles, where the implantation of embryos with the correct number of chromosomes is critical for achieving successful pregnancy outcomes. This review underscores its role in enhancing implantation rates and reducing pregnancy loss by assessing the evolution, methodology, clinical applications, efficacy, and challenges associated with ERA. Key findings highlight ERA's superior accuracy in identifying the window of implantation compared to traditional methods, resulting in improved clinical outcomes in assisted reproductive technology (ART) cycles. Despite its benefits, the review acknowledges challenges such as cost, accessibility, and the need for standardization. Recommendations for clinical practice emphasize the integration of ERA into routine ART protocols, comprehensive patient counseling, and the importance of multidisciplinary collaboration. The review outlines promising prospects, including technological advancements to make ERA more cost-effective, the development of refined gene expression profiles, and the potential integration with other emerging ART technologies. Further research directions include long-term studies on the outcomes of ERA-guided pregnancies and exploring its application in cases of recurrent implantation failure and unexplained infertility. Overall, ERA represents a significant advancement in reproductive medicine, offering a personalized approach to embryo transfer timing that can significantly improve the success rates of euploid embryo transfers.

## Introduction and background

The Endometrial Receptivity Array (ERA) is a cutting-edge molecular diagnostic tool designed to assess the endometrium's receptive state during the implantation window. Unlike traditional methods, which rely on histological dating or ultrasound evaluation, ERA analyzes the gene expression profile of endometrial tissue to determine the optimal timing for embryo transfer in assisted reproductive technology (ART) cycles [1]. By examining the expression levels of specific genes involved in endometrial receptivity, ERA provides personalized insights into the receptivity status of the endometrium, allowing for the precise timing of embryo transfer to maximize the chances of successful implantation and pregnancy [2]. In euploid embryo transfer cycles, where embryos with the correct number of chromosomes are transferred, the receptivity of the endometrium plays a critical role in determining the success of implantation and subsequent pregnancy [3].

The window of implantation (WOI) is a brief period during the menstrual cycle when the endometrium is optimally receptive to embryo implantation. Any deviation from the ideal receptivity window can significantly impact implantation rates and the likelihood of achieving a viable pregnancy. Therefore, accurately assessing endometrial receptivity is essential for optimizing the timing of embryo transfer and improving the outcomes of euploid embryo transfer cycles [4].

This review aims to provide a comprehensive overview of ERA and its application in euploid embryo transfer cycles. By synthesizing existing literature, clinical data, and research findings, this review aims to elucidate the methodology, clinical significance, efficacy, challenges, and prospects of ERA in the ART context. Additionally, this review seeks to highlight the potential impact of ERA on clinical practice, patient outcomes, and the field of reproductive medicine.

## Review

Endometrial receptivity in euploid embryo transfer cycles

Definition of Euploid Embryo Transfer

A healthy and genetically normal embryo is carefully placed into the uterus during the process of euploid embryo transfer. Euploid embryos possess the precise number of chromosomes necessary for a successful pregnancy. This method is considered optimal as it greatly diminishes the risks associated with miscarriage and chromosomal abnormalities in the developing fetus [5-7]. During euploid embryo transfer, embryos are meticulously selected through preimplantation genetic testing (PGT) utilizing cutting-edge technologies such as next-generation sequencing. This meticulous selection process ensures that only embryos with the correct chromosome count are chosen for transfer. Consequently, the chances of transferring aneuploid or mosaic embryos, ones that could result in miscarriage or birth defects, are substantially reduced [5-7]. Euploid embryo transfer holds particular significance for patients grappling with advanced maternal age, recurrent pregnancy loss, or recurrent implantation failure (RIF). For these individuals, this approach has demonstrated remarkable potential in enhancing pregnancy outcomes [5-7].

Factors Affecting Endometrial Receptivity

Maternal age represents a significant factor in female infertility, often overlooked in discussions. The endometrium undergoes cellular, molecular, and hormonal changes with advancing age. These alterations can detrimentally affect receptivity, diminishing implantation and pregnancy rates [8]. Polycystic ovary syndrome (PCOS) patients present distinctive challenges in terms of endometrial receptivity compared to their counterparts without the syndrome. Individuals with PCOS often exhibit disruptions in endometrial receptivity markers. These alterations manifest in various facets, including gene expression, energy metabolism, and the endocrine environment. Consequently, the impaired endometrial receptivity observed in PCOS patients correlates with reduced embryo implantation rates [9]. The impact of stress and hormonal imbalances on endometrial receptivity is profound. Fertility-related stress and anxiety, coupled with hormonal dysregulation marked by elevated cortisol and prolactin levels, can significantly compromise endometrial receptivity. These disturbances affect crucial aspects such as blood flow, insulin sensitivity, and the expression of critical receptors and growth factors, ultimately impairing embryo implantation [10].

**Figure 1:**
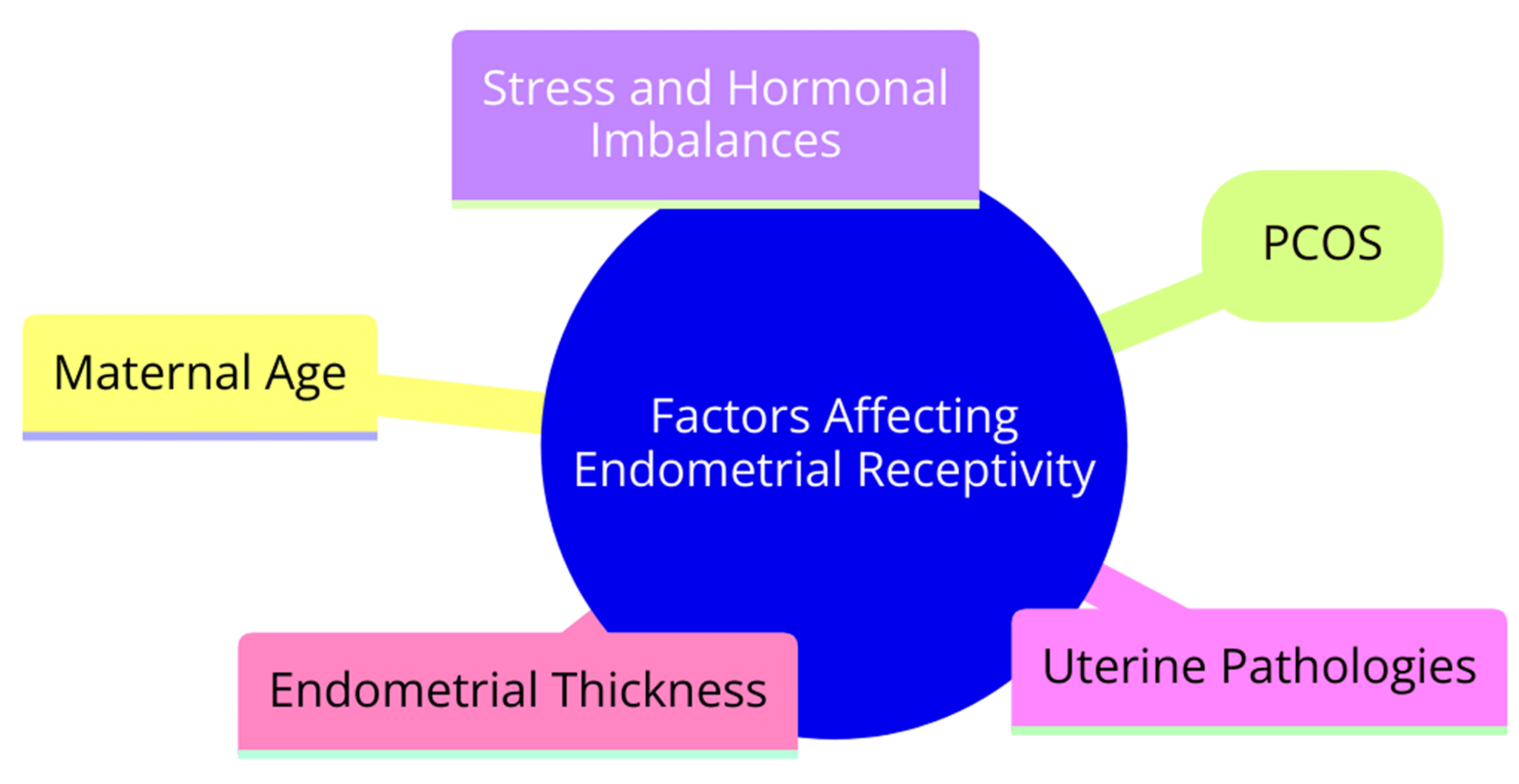
Factors affecting endometrial receptivity Image credit: Dr Medhavi Sharma

Uterine pathologies encompass a range of conditions, each capable of disrupting the endometrium physically or biochemically. Conditions such as uterine polyps, fibroids, septa, adhesions, and endometriosis pose significant challenges to endometrial receptivity. Their presence compromises the optimal environment for successful embryo implantation, thus impacting overall pregnancy outcomes [11]. Endometrial thickness is a critical determinant of receptivity, with both extremes, very thin and very thick endometrium, associated with reduced implantation and pregnancy rates in in-vitro fertilization (IVF) cycles. This underscores the pivotal role of achieving optimal endometrial development for facilitating successful embryo implantation and subsequent pregnancy [11]. Factors affecting endometrial receptivity are shown in Figure 1.

Significance of Timed Endometrial Receptivity in Euploid Embryo Transfer

The precise timing of endometrial receptivity in euploid embryo transfer cycles holds significant importance in ART. Research indicates that aligning the timing of embryo transfer with the optimal window of endometrial receptivity, as determined by tests such as ERA, can significantly influence the success rates of embryo implantation and subsequent live births in patients undergoing IVF with euploid embryos [12-14].

Endometrial receptivity testing aims to pinpoint the ideal WOI tailored to each patient's characteristics through molecular analysis of the endometrium. This personalized approach aids in determining the most opportune moment for embryo transfer, potentially enhancing outcomes, particularly in patients with a history of RIF despite possessing known euploid embryos [12,14]. The research underscores the critical role of synchronizing the embryo and endometrium for successful implantation.

The ERA test is adept at identifying instances of a displaced WOI, where the endometrium may not be receptive at the standard transfer time, consequently leading to unsuccessful transfers. Adjusting the timing of embryo transfer based on receptivity testing results offers a means to rectify this issue and potentially elevate the likelihood of achieving a successful pregnancy [12]. While the ERA test has demonstrated promise in improving outcomes, particularly in specific patient cohorts such as those with RIF, its universal effectiveness across all euploid embryo transfer cycles remains a topic of ongoing research and discussion. Further investigations, including adequately powered randomized clinical trials, are imperative to understand the impact of timed endometrial receptivity on the success rates of euploid embryo transfers in the context of IVF treatments [13,14].

Evolution of endometrial receptivity assessment

Historical Context and Traditional Methods

The evolution of assessing endometrial receptivity has witnessed remarkable progressions, transitioning from conventional methodologies to sophisticated molecular diagnostic tools such as ERA [14-16]. Historically, endometrial receptivity assessment relied on traditional techniques such as endometrial histology, which posed limitations in accuracy and reproducibility [15]. Evaluating the endometrium involved observing morphological changes throughout the menstrual cycle, as first delineated by Noyes et al. in 1975 [2]. However, these conventional methods proved insufficiently sensitive or specific, exhibiting poor inter-observer consistency [15]. Notably, the endometrial biopsy, a vital component of these techniques, was both time- and labor-intensive, necessitating substantial expertise for precise interpretation [14]. The advent of ERA in 2011 heralded a significant paradigm shift in endometrial receptivity assessment [14,16]. Utilizing a 236-gene expression profile, ERA identifies the optimal timing for embryo transfer, presenting a more refined and dependable approach than traditional histological evaluations [14-16].

Emergence of ERA

ERA has emerged as a promising diagnostic tool for evaluating the endometrial receptivity crucial for supporting blastocyst implantation. This innovative method scrutinizes the expression patterns of 248 molecular genes to ascertain the optimal timing for embryo transfer [16-18]. ERA offers several advantages over conventional approaches, such as endometrial histology, boasting enhanced accuracy and reproducibility [16,17]. Its utility is particularly pronounced for women grappling with a history of RIF or multiple unsuccessful IVF cycles, facilitating personalized embryo transfer (pET) by pinpointing the ideal timing for progesterone administration before embryo transfer [17,18]. The ERA procedure entails an endometrial biopsy, where a minute sample of the uterine lining undergoes genetic analysis to delineate the optimal timing for hormone administration. The resulting outcomes are typically categorized as either "receptive" or "non-receptive" [17,18]. When the results indicate non-receptivity, a subsequent ERA test is often recommended to ascertain the WOI [17].

Despite its potential benefits, current evidence suggests that ERA may not yield significant enhancements in reproductive outcomes within euploid embryo transfer cycles, irrespective of whether the patients are from the general infertile population or possess a history of previous failed embryo transfers [17,19]. A meta-analysis revealed that ERA exerted no discernible impact on reproductive outcomes in frozen embryo transfer cycles [19]. Nevertheless, a compelling case report underscored ERA's ability to identify a displaced WOI in patients with a track record of euploid blastocyst implantation failure. This finding suggests that pET employing a tailored progesterone protocol may yield improved outcomes in these scenarios [18].

Advantages of Conventional Methods

ERA stands out for its precision in evaluating endometrial receptivity, analyzing the expression patterns of 248 molecular genes, which is a method far superior in accuracy compared to traditional approaches such as endometrial histology [2,20]. Moreover, the ERA test results boast complete reproducibility, starkly contrasting the inconsistency often observed in histological evaluations [2,21]. This reliability is particularly valuable, given the critical role of timing in embryo transfer success. One of ERA's notable strengths is its ability to provide a personalized approach, identifying a patient-specific WOI for optimal embryo transfer timing feature immensely beneficial for individuals grappling with RIF [2,21]. This tailored approach enhances the likelihood of successful embryo implantation, potentially addressing previous challenges encountered in standard protocols.

The objectivity of ERA is another noteworthy attribute, offering an unbiased assessment of endometrial receptivity. In contrast, histological evaluation utilizing the Noyes criteria has faced scrutiny regarding its accuracy and reproducibility [22]. By delving into gene expression profiles, ERA delves deeper into the molecular mechanisms underpinning endometrial receptivity, yielding insights unattainable through conventional methods [20,22]. Though the evidence regarding ERA's ability to improve reproductive outcomes is mixed, some studies suggest promising prospects, particularly in patient populations with a history of euploid blastocyst implantation failure. The pET guided by ERA can potentially enhance success rates in these specific cases [20,21]. Continued research and clinical validation are essential to fully elucidate the extent of ERA's impact on reproductive outcomes and its applicability across diverse patient cohorts.

Methodology of ERA

Sample Collection and Processing

Conducting an ERA begins with collecting a small sample of endometrial tissue, a procedure typically performed on a specific day as determined by the physician. This timing often aligns with LH+7 in a natural cycle or P+5 in a hormone replacement therapy (HRT) cycle [17,20,23]. Subsequently, RNA extraction and amplification are carried out on the obtained endometrial sample using polymerase chain reaction (PCR) techniques [24]. Following this, the amplified RNA is subjected to microarray analysis, where it is hybridized to a customized microarray chip containing probes targeting the 248 genes associated with endometrial receptivity. This process enables the measurement of gene expression levels [17,20]. The obtained gene expression data undergo computational analysis utilizing a predictor algorithm. This algorithm compares the transcriptomic profile of the sample to a reference database comprising receptive and non-receptive endometrial samples. Based on this comparison, the algorithm categorizes the sample as either receptive or non-receptive, with non-receptive samples further classified as pre-receptive or post-receptive. The ERA test has demonstrated high sensitivity (0.99758) and specificity (0.8857) for diagnosing endometrial receptivity. Ultimately, the ERA test aims to ascertain the optimal time window for embryo transfer by assessing the expression of the 248 molecular genes associated with endometrial receptivity [24].

Gene Expression Profiling

Gene expression profiling is a potent technique within molecular biology, enabling the simultaneous measurement of the activity of numerous genes. This method offers a comprehensive insight into cellular function, allowing researchers to differentiate between cells based on their distinct gene expression patterns. These patterns aid in various endeavors, including identifying actively dividing cells and comprehending cellular responses to specific treatments. Gene expression profiling encompasses technologies such as DNA microarrays and RNA-Seq, which facilitate the analysis of gene expression levels and sequences [25]. Researchers spanning diverse fields harness gene expression profiling to glean insights into biological processes, explore tissue-specific functions, and delve into disease mechanisms. By quantifying the relative abundance of mRNA expressed under different experimental conditions, researchers can discern alterations in gene expression indicative of responses to stimuli, disease states, or other conditions. This wealth of information catalyzes the development of new hypotheses, validation of existing theories, and exploration of potential diagnostic applications. Gene expression profiling is a linchpin in advancing our comprehension of cellular behavior, disease progression, and responses to pharmaceutical interventions. By furnishing a detailed snapshot of gene activity across varied biological contexts, this technique paves the way for substantial advancements in biomedical research [26].

Clinical applications of ERA

pET Timing

The pET, guided by endometrial receptivity testing, endeavors to enhance reproductive outcomes by pinpointing the optimal WOI tailored to each patient's physiology. However, in a randomized clinical trial, pET guided by endometrial receptivity testing failed to yield a significant improvement in live birth rates compared to standard embryo transfer timing [13]. Notably, the live birth rate stood at 63.5% in the pET group, marginally higher than the 61.2% observed in the standard timing group. Conversely, a retrospective study unveiled contrasting findings, indicating that pET based on endometrial receptivity results correlated with elevated pregnancy rates compared to standard timing, both for the initial embryo transfer (72.5% vs. 54.3%) and cumulatively (93.6% vs. 79.7%) [14]. Moreover, live birth rates demonstrated an upward trend with pET utilization. Notably, for patients exhibiting a displaced WOI as identified by endometrial receptivity testing, pET conducted at the optimal time predicted by the test yielded reproductive outcomes akin to those with a receptive WOI [27]. This suggests that pET possesses the potential to surmount challenges associated with a displaced WOI. In modified natural frozen embryo transfer (nFET) cycles, the embryo transfer typically occurs seven days after hCG administration (hCG+7) [14]. However, the optimal timing may vary depending on the endometrial receptivity profile. This underscores the necessity for individualized approaches in embryo transfer timing to maximize the chances of successful implantation and subsequent pregnancy.

Improving Implantation Rates

Improving implantation rates in IVF cycles remains paramount for optimizing reproductive outcomes. Extensively studied for its potential impact on implantation rates, ERA has garnered attention within the scientific community. However, research findings suggest that ERA may not significantly enhance reproductive outcomes in euploid embryo transfer cycles. Indeed, a systematic review revealed that ERA-guided embryo transfers failed to yield optimized reproductive outcomes [21]. Despite the allure of pET guided by ERA, studies have cast doubt on its efficacy in improving implantation rates and sustaining ongoing pregnancies, particularly in patients grappling with RIF [4]. While specific studies have hinted at the potential benefits of ERA in enhancing pregnancy and implantation rates among women with RIF, the overall evidence remains inconclusive. For instance, while one study documented a surge in implantation and ongoing pregnancy rates in RIF patients subjected to ERA-guided pET, conflicting results from other studies have emerged, suggesting that ERA-guided transfers may not consistently translate into improved reproductive outcomes [28]. The impact of ERA on enhancing implantation rates in IVF cycles remains a subject of ongoing investigation. While select studies propose potential benefits in specific patient populations, the need for further research persists to ascertain the consistent effectiveness of ERA in augmenting implantation rates across diverse IVF scenarios. Continued exploration and validation are essential to elucidate the true potential of ERA and its applicability in optimizing reproductive outcomes in clinical settings.

Reducing Pregnancy Loss

The utilization of ERA in clinical practice has yielded diverse outcomes concerning the reduction of pregnancy loss. While specific studies suggest that ERA may not significantly enhance pregnancy outcomes in patients with a favorable prognosis [14], other research indicates a potential enhancement of pregnancy and implantation rates by nearly 20% in women grappling with RIF [2]. In instances of RIF characterized by multiple failed embryo transfers, ERA emerges as a promising tool for improving outcomes by guiding pET strategies based on the endometrial receptivity status [29]. This tailored approach has been associated with heightened reproductive performance, including augmented ongoing pregnancy rates and implantation rates, particularly in challenging cases such as RIF [19]. Consequently, the clinical integration of ERA, particularly in scenarios of RIF, holds promise as a strategy to mitigate pregnancy loss by optimizing the timing of embryo transfer based on the endometrial receptivity status. However, to fully elucidate the impact of ERA on reducing pregnancy loss across diverse patient populations undergoing ART, further research endeavors and larger-scale studies are imperative [30]. Continued investigation is essential for validating the efficacy and applicability of ERA in clinical settings, ultimately paving the way for improved reproductive outcomes and enhanced patient care.

Efficacy and clinical outcomes

Meta-Analyses and Clinical Studies

Several studies and reviews have contributed to our understanding of ERA and its impact on reproductive outcomes, particularly in frozen embryo transfer cycles. A systematic review and meta-analysis concluded that ERA before frozen embryo transfer cycles does not improve live birth rates [19]. Additionally, another review article highlighted the clinical applications of ERA, acknowledging its provision of a personalized approach to embryo transfer and emphasizing the need for further investigation into its impact on pregnancy outcomes [14]. A retrospective study examined the use of ERA to guide pET after a failed transfer attempt and found no significant improvement in live birth rates compared to frozen embryo transfers without ERA guidance [28]. However, within a subgroup of patients with a history of euploid blastocyst implantation failure, 22.5% exhibited a displaced WOI identified by ERA. For these patients, pET using a modified progesterone protocol may enhance outcomes of subsequent embryo transfer attempts, although more extensive randomized studies are required for validation [31].

Furthermore, another systematic review concluded that ERA failed to optimize reproductive outcomes in embryo-endometrial transfer (EET) cycles, regardless of whether in the general infertile population or patients with a history of previous failed embryo transfers [19]. While ERA can identify a displaced WOI in certain patients, its consistent ability to improve reproductive outcomes in EET cycles remains to be determined based on current evidence. Therefore, larger, well-designed studies are necessary to definitively ascertain the role of ERA in optimizing EET success.

Success Rates Compared to Conventional Methods

The success rates of device-assisted enteroscopy techniques, such as double-balloon enteroscopy (DBE) and single-balloon enteroscopy (SBE), present variations compared to conventional methods such as video capsule endoscopy (VCE). The technical success rate for colonoscopy completion within this realm ranges from 85% to 98%, with additional findings reported in 14%-45% of cases [32]. Studies on DBE have consistently reported diagnostic rates within the range of 43%-81%, alongside treatment success rates ranging from 43%-84% [33,34]. A meta-analysis yielded no significant difference in the overall diagnostic yield between VCE (57%) and balloon-assisted enteroscopy (BAE) (62%) [34]. However, when both antegrade and retrograde BAEs were employed, the yield of BAE surpassed that of VCE (88% vs. 46%) [34]. Furthermore, in a study utilizing DBE to complete previously failed colonoscopies, successful cecal intubation was accomplished in 88%-100% of patients [34]. A prospective study on motorized spiral enteroscopy unveiled a technical success rate of 94% and sufficient insertion depth in 89% of cases [35]. These findings underscore the diverse efficacy and utility of device-assisted enteroscopy techniques in diagnosing and treating gastrointestinal conditions, offering valuable insights into their comparative effectiveness against conventional methods.

Long-Term Follow-Up Data

Long-term follow-up data regarding the efficacy of ERA in enhancing reproductive outcomes for euploid embryo transfer (EET) cycles remains limited. A retrospective study noted that utilizing ERA to guide pET post-failed transfer attempts did not yield improved live birth rates compared to frozen embryo transfers without ERA guidance. However, the study did not specify the duration of the follow-up period [31]. Within a cohort of patients with a history of euploid blastocyst implantation failure, 22.5% exhibited a displaced WOI diagnosed by ERA. For these individuals, employing pET with a modified progesterone protocol may enhance the outcomes of subsequent EET. Notably, the study solely reported results from the initial pET cycle, needing long-term follow-up data [19]. Moreover, a systematic review and meta-analysis concluded that the routine use of ERA before frozen embryo transfer cycles did not improve live birth rates. However, the studies included in the analysis had follow-up limited to a single embryo transfer cycle [14].

Similarly, a retrospective cohort study revealed that employing ERA to guide pET post-failed transfer attempts was linked to lower live birth rates than frozen embryo transfers without ERA guidance. Yet, similar to previous studies, this investigation monitored patients for only a single pET cycle, offering limited insight into long-term outcomes [28]. These findings underscore the need for comprehensive, long-term follow-up studies to elucidate the enduring impact of ERA on reproductive outcomes in EET cycles. Such investigations would provide valuable insights into the efficacy and utility of ERA in clinical practice, guiding informed decision-making regarding its integration into assisted reproductive treatments.

Future directions and potential developments

Integration With Other ART

The integration of AI in ART, particularly in automation, is geared toward enhancing the precision, standardization, and accessibility of ART procedures. The field anticipates heightened efficiency and personalized treatment pathways by infusing AI into critical stages of the ART process, including patient identification, gamete/embryo selection, endometrial evaluation, and cryopreservation [36-38]. Envisioning the future of ART entails a transformative shift driven by the amalgamation of AI, automation, and other cutting-edge technologies. Innovations such as "IVF in a box" harness microfluidics, robotics, and AI to devise fully automated and intelligent devices for IVF treatments, hinting at a potential revolution in infertility treatments [38]. AI's role in refining current clinical practices within ART extends to enhancing predictive capacities, streamlining workflows, and elevating patient and healthcare provider outcomes. The overarching aim is not to supplant human expertise but to complement it, fostering more precise decision-making and standardized processes in ART [37,38]. However, alongside the promise of integrating AI with ART come challenges pertinent to implementing innovative technologies in clinical settings. Bridging the chasm between research and clinical application is imperative to standardize the utilization of automation and AI in bolstering ART outcomes. Moreover, ensuring accessibility and affordability for patients remains a pivotal consideration in realizing the full potential of these advancements [38].

Technological Advances in Endometrial Profiling

Studies in the field have leveraged DNA microarrays and oligonucleotide microarray technology to delve into global changes in gene expression throughout the endometrial receptivity process. These methodologies have empowered researchers to pinpoint immune modulatory genes, adhesion molecules, genes implicated in oxidative stress, and cytoskeletal proteins, thereby elucidating the intricate molecular mechanisms governing endometrial receptivity [39]. The advent of a highly quantitative TAC-seq method has ushered in a new era of endometrial dating and the identification of transcriptome biomarkers linked to endometrial receptivity. This innovative analytical pipeline facilitates dynamic and sensitive detection of selected transcriptome biomarkers, furnishing a quantitative and precise forecast of endometrial receptivity status [40]. Endometrial immune profiling has emerged as a pivotal strategy in tailoring personalized care within assisted reproductive medicine. By quantifying immune biomarkers in endometrial biopsies and scrutinizing the immune milieu of the uterus, this approach endeavors to optimize pregnancy outcomes through tailored treatment strategies predicated on the immune profile of the endometrium [41]. The application of multi-omics profiling, encompassing genomics and immunological analyses, has provided insights into the distinct microenvironment of endometrial cancer. By delineating immune cell abundance, mutated genes, and copy number alterations across different clusters, this approach enhances comprehension of the immunophenotype of endometrial cancer and its prognostic implications [42].

Implications for Precision Medicine in Reproductive Health

The implications of precision medicine in reproductive health are profound and offer the prospect of advancing personalized approaches within the field. Precision medicine, which entails tailoring treatments based on individual characteristics such as genetic profiles, lifestyle, and environmental factors, stands poised to revolutionize reproductive medicine. By embracing the principles of precision medicine, practitioners in reproductive health can augment various facets of care, encompassing diagnosis, treatment planning, and embryo identification, thereby facilitating more efficacious and personalized interventions for individuals contending with fertility challenges [43,44]. In reproductive medicine, precision medicine presents an opportunity to gather comprehensive information on a patient's health history, familial background, lifestyle choices, and genotype, enabling the development of tailored treatment protocols. This personalized approach could enhance the effectiveness and efficiency of ART by transitioning from a one-size-fits-all treatment paradigm to one that accounts for individual patient attributes to yield superior outcomes.

Moreover, integrating genetic data and extensive data analysis can yield deeper insights into the underlying mechanisms of infertility, fostering the development of more targeted and efficacious interventions [43]. Moreover, the application of precision medicine in reproductive health has the potential to tackle the escalating complexity of fertility disorders and the myriad underlying causes. By prioritizing precise diagnosis, meticulous embryo identification, and optimization of treatment procedures, precision medicine can surmount obstacles in reproductive medicine and chart a course toward more customized and successful interventions. Ultimately, the integration of precision medicine concepts in reproductive health harbors the potential to ameliorate patient outcomes, refine treatment modalities, and propel the field toward more individualized and efficacious care for individuals grappling with infertility challenges [45].

## Conclusions

In conclusion, ERA has emerged as a pivotal innovation in reproductive medicine, particularly in optimizing the timing of euploid embryo transfers. By leveraging gene expression profiling, ERA offers a precise and personalized approach to identifying the optimal WOI, thereby significantly enhancing implantation and pregnancy rates. Clinical studies underscore its efficacy, showing improved outcomes compared to traditional methods. Despite its benefits, challenges such as cost, accessibility, and the need for standardized interpretation persist. Comprehensive training and multidisciplinary collaboration are essential to integrate ERA effectively into clinical practice. Future advancements in technology and further research are expected to make ERA more accessible and enhance its accuracy. Additionally, exploring its applications in broader contexts and combining it with other emerging technologies could revolutionize personalized treatment in ART. Ultimately, ERA represents a substantial step toward achieving higher success rates and better patient outcomes in ART.

## References

[1] Mahajan N (2015). Endometrial receptivity array: clinical application. J Hum Reprod Sci.

[2] Blanco-Breindel MF, Singh M, Kahn J (2023). Endometrial receptivity. StatPearls [Internet].

[3] Reshef EA, Robles A, Hynes JS, Turocy JM, Forman EJ (2022). A review of factors influencing the implantation of euploid blastocysts after in vitro fertilization. F&S Reviews.

[4] Enciso M, Aizpurua J, Rodríguez-Estrada B (2021). The precise determination of the window of implantation significantly improves ART outcomes. Sci Rep.

[5] Singh PN, Pathak AM, Singh P, Desai M (2022). Selecting euploid embryos for transfer by preimplantation genetic testing with the help of next-generation sequencing in poor prognosis patients: a retrospective cohort analysis. J Hum Reprod Sci.

[6] (2024). Euploid embryo vs. aneuploid embryo. https://triofertility.com/euploid-embryo-vs-aneuploid-embryo/.

[7] (2024). Is my embryo normal, abnormal or mosaic?. https://triofertility.com/is-my-embryo-normal-abnormal-or-mosaic/.

[8] Pathare AD, Loid M, Saare M (2023). Endometrial receptivity in women of advanced age: an underrated factor in infertility. Hum Reprod Update.

[9] Bai X, Zheng L, Li D, Xu Y (2021). Research progress of endometrial receptivity in patients with polycystic ovary syndrome: a systematic review. Reprod Biol Endocrinol.

[10] Vujović S, Ivovic M, Tančić-Gajić M (2021). Detection and treatment of some endometrial receptivity disorders - a way to improve fertility rates. GREM.

[11] Revel A (2012). Defective endometrial receptivity. Fertil Steril.

[12] Girona R (2024). Endometrial receptivity array (ERA): the definitive clinical trial?. IVIRMA Innovation.

[13] Doyle N, Jahandideh S, Hill MJ, Widra EA, Levy M, Devine K (2022). Effect of timing by endometrial receptivity testing vs standard timing of frozen embryo transfer on live birth in patients undergoing in vitro fertilization: a randomized clinical trial. JAMA.

[14] Rubin SC, Abdulkadir M, Lewis J, Harutyunyan A, Hirani R, Grimes CL (2023). Review of endometrial receptivity array: a personalized approach to embryo transfer and its clinical applications. J Pers Med.

[15] (2024). Endometrial receptivity assessment (ERA). https://www.fcionline.com/article/endometrial-receptivity-assessment-era/.

[16] (2024). Endometrial receptivity analysis (ERA). http://www.medparkhospital.com/en-US/disease-and-treatment/endometrial-receptivity-analysis.

[17] PhD KB (2024). What is an endometrial receptivity array (ERA)?. https://pathfertility.com/what-is-an-endometrial-receptivity-array-era/#:~:text=An%20endometrial%20receptivity%20array%20(ERA)%20is%20a%20type%20of%20test,implantation%20failure%20following%20embryo%20transfer..

[18] (2024). Endometrial receptivity array: who should opt for ERA. https://www.indiraivf.com/blog/endometrial-receptivity-array-era..

[19] Arian SE, Hessami K, Khatibi A, To AK, Shamshirsaz AA, Gibbons W (2023). Endometrial receptivity array before frozen embryo transfer cycles: a systematic review and meta-analysis. Fertil Steril.

[20] (2024). Decoding fertility: understanding the endometrial receptivity array. https://nimaaya.com/blog/endometrial-receptivity-array/.

[21] Mei Y, Wang Y, Ke X, Liang X, Lin Y, Wang F (2023). Does endometrial receptivity array improve reproductive outcomes in euploid embryo transfer cycles? A systematic review. Front Endocrinol (Lausanne).

[22] Lu Y, Mao X, He Y, Wang Y, Sun Y (2024). Efficacy of endometrial receptivity testing for recurrent implantation failure in patients with euploid embryo transfers: study protocol for a randomized controlled trial. Trials.

[23] (2024). Endometrial receptivity array (ERA): endometrial biopsy. https://fertilitynj.com/services/female-fertility-evaluation/era-endometrial-biopsy/.

[24] Jain DS (2024). What is endometrial receptivity array (ERA) test for IVF and why is it important?. AIIM.

[25] Alberts B, Johnson A, Lewis J (2002). Molecular Biology of the Cell. Molecular Biology of the Cell. 4th edition. Garland Science.

[26] Chengalvala MV, Chennathukuzhi VM, Johnston DS, Stevis PE, Kopf GS (2007). Gene expression profiling and its practice in drug development. Curr Genomics.

[27] Li N, Zhang Y, Li R (2024). Personalized embryo transfer guided by rsERT improves pregnancy outcomes in patients with repeated implantation failure. Front Med (Lausanne).

[28] Cozzolino M, Diáz-Gimeno P, Pellicer A, Garrido N (2022). Use of the endometrial receptivity array to guide personalized embryo transfer after a failed transfer attempt was associated with a lower cumulative and per transfer live birth rate during donor and autologous cycles. Fertil Steril.

[29] Zhang WB, Li H, Lu X (2022). The clinical efficiency of transcriptome-based endometrial receptivity assessment (Tb-ERA) in Chinese patients with recurrent implantation failure (RIF): a study protocol for a prospective randomized controlled trial. Contemp Clin Trials Commun.

[30] Franasiak JM, Alecsandru D, Forman EJ (2021). A review of the pathophysiology of recurrent implantation failure. Fertil Steril.

[31] Tan J, Kan A, Hitkari J (2018). The role of the endometrial receptivity array (ERA) in patients who have failed euploid embryo transfers. J Assist Reprod Genet.

[32] Hanscom M, Stead C, Feldman H, Marya NB, Cave D (2022). Video capsule endoscopy and device-assisted enteroscopy. Dig Dis Sci.

[33] Keum B, Chun HJ (2011). Capsule endoscopy and double balloon enteroscopy for obscure gastrointestinal bleeding: which is better?. J Gastroenterol Hepatol.

[34] Elena RM, Riccardo U, Rossella C, Bizzotto A, Domenico G, Guido C (2012). Current status of device-assisted enteroscopy: technical matters, indication, limits and complications. World J Gastrointest Endosc.

[35] Falt P, Urban O (2023). Motorized spiral enteroscopy - a prospective analysis of 82 procedures at a single tertiary center. Scand J Gastroenterol.

[36] Letterie G (2023). Artificial intelligence and assisted reproductive technologies: 2023. Ready for prime time? Or not. Fertil Steril.

[37] Embryologist PP (2024). Automation in ART: shaping the future of treatments for infertility. https://www.esco-medical.com/news/automation-in-art-shaping-the-future-of-treatments-for-infertility.

[38] Abdullah KA, Atazhanova T, Chavez-Badiola A, Shivhare SB (2023). Automation in ART: paving the way for the future of infertility treatment. Reprod Sci.

[39] Riesewijk A, Martín J, van Os R (2003). Gene expression profiling of human endometrial receptivity on days LH+2 versus LH+7 by microarray technology. Mol Hum Reprod.

[40] Meltsov A, Saare M, Teder H (2023). Targeted gene expression profiling for accurate endometrial receptivity testing. Sci Rep.

[41] Lédée N, Petitbarat M, Prat-Ellenberg L (2020). Endometrial immune profiling: a method to design personalized care in assisted reproductive medicine. Front Immunol.

[42] Cai Y, Chang Y, Liu Y (2019). Multi-omics profiling reveals distinct microenvironment characterization of endometrial cancer. Biomed Pharmacother.

[43] Zhang PY, Yu Y (2019). Precise personalized medicine in gynecology cancer and infertility. Front Cell Dev Biol.

[44] Lamichhane P, Agrawal A (2023). Precision medicine and implications in medical education. Ann Med Surg (Lond).

[45] de Santiago I, Polanski L (2022). Data-driven medicine in the diagnosis and treatment of infertility. J Clin Med.

